# A multispecies polyadenylation site model

**DOI:** 10.1186/1471-2105-14-S2-S9

**Published:** 2013-01-21

**Authors:** Eric S Ho, Samuel I Gunderson, Siobain Duffy

**Affiliations:** 1 Department of Molecular Genetics, Microbiology and Immunology, University of Medicine and Dentistry of New Jersey-Robert Wood Johnson Medical School, Piscataway, New Jersey, USA; 2 Department of Molecular Biology and Biochemistry, Rutgers University, New Brunswick, New Jersey, USA; 3 Department of Ecology, Evolution and Natural Resources, Rutgers University, New Brunswick, NJ, USA

## Abstract

**Background:**

Polyadenylation is present in all three domains of life, making it the most conserved post-transcriptional process compared with splicing and 5'-capping. Even though most mammalian poly(A) sites contain a highly conserved hexanucleotide in the upstream region and a far less conserved U/GU-rich sequence in the downstream region, there are many exceptions. Furthermore, poly(A) sites in other species, such as plants and invertebrates, exhibit high deviation from this genomic structure, making the construction of a general poly(A) site recognition model challenging. We surveyed nine poly(A) site prediction methods published between 1999 and 2011. All methods exploit the skewed nucleotide profile across the poly(A) sites, and the highly conserved poly(A) signal as the primary features for recognition. These methods typically use a large number of features, which increases the dimensionality of the models to crippling degrees, and typically are not validated against many kinds of genomes.

**Results:**

We propose a poly(A) site model that employs minimal features to capture the essence of poly(A) sites, and yet, produces better prediction accuracy across diverse species. Our model consists of three dior-trinucleotide profiles identified through principle component analysis, and the predicted nucleosome occupancy flanking the poly(A) sites. We validated our model using two machine learning methods: logistic regression and linear discriminant analysis. Results show that models achieve 85-92% sensitivity and 85-96% specificity in seven animals and plants. When we applied one model from one species to predict poly(A) sites from other species, the sensitivity scores correlate with phylogenetic distances.

**Conclusions:**

A four-feature model geared towards small motifs was sufficient to accurately learn and predict poly(A) sites across eukaryotes.

## Background

Nearly all eukaryotic messenger RNA (mRNA) carries a long series of adenine at the 3' end called the polyadenylation (poly(A)) tail. The molecular process synthesizing the poly(A) tail is called polyadenylation. Eukaryotic polyadenylation was first reported more than half a century ago [1]. Since then, tremendous progress has been made in elucidating the mechanism, regulation, protein factors, and related biological functions. Although polyadenylated transcripts in prokaryotes were first identified since 1975 [2,3], the majority of studies focus on eukaryotes and their DNA viruses, probably due to the obstacles of isolating unstable prokaryotic transcripts. More recently, polyadenylation has been studied in Archaea [4-6] and in organelles: the chloroplast [7-10], and mitochondria [11,12]. The prevalence of polyadenylation across all three domains of life signifies a long evolutionary history in which varied complexity and additional functions have been selected by diverse species.

Polyadenylation consists of two tandem enzymatic reactions: the cleavage of a nascent mRNA from the elongating RNA polymerase, followed by the non-template synthesis of a poly(A) tail that varies in length between speices. A typical eukaryotic poly(A) site is characterized by three cis-elements. The first element lies where the pre-mRNA is cut off from the RNA polymerase at the pre-mRNA's 3'-most exon: the cleavage site. The second element is a highly conserved hexanucleotide, namely the poly(A) signal. The majority of poly(A) signals are located ~20 nts upstream from the cleavage sites. 66% and 16% of mammalian transcripts contain AAUAAA and AUUAAA, respectively [13,14], making the canonical poly(A) signal AWUAAA (W stands for 'A' or 'U'). The third element is named the downstream element (DSE) which is located at ~10-15 nts downstream from the cleavage site. In contrast to the poly(A) signal, no consensus sequence has been found in the DSE among animals except that it is enriched mainly with 'U' and sprinkled with 'G'. Therefore the DSE is known as U/GU-rich region. Although cis-elements are short and variable, polyadenylation takes place precisely (± 5 nts) at the same location (or locations in the case of alternative polyadenylation) of a gene. Moreover, even though all genes within a species are processed by the same set of core polyadenylation factors, two poly(A) sites rarely resemble each other [15]. The functionally conserved but sequence-variable poly(A) sites not only challenge the identification of definitive features for recognition, but also present an intriguing case study for the understanding of the evolution of non-coding regions in different species.

We present an improved poly(A) site model that distinguishes itself from existing models in four ways. 1) Instead of choosing features haphazardly, we use principal component analysis (PCA) to identify the localization of cis-elements without presuming what they are. 2) Our four feature model uses fewer features than existing methods (Table S1 of Additional file 1), which use between six and over 5,000 features [16], and achieves superior prediction accuracy. The rationale of taking a parsimonious approach in feature selection is to circumvent the dimensionality curse [17,18], but our simple model also requires a smaller training dataset as a result. 3) Despite the highly variable poly(A) site cis-elements, the poly(A) complex is still able to cleave the transcript at the same position. We believe the poly(A) site is marked by more information than just sequence elements, such as peculiar chromatin structure [19]. Therefore, we have incorporated nucleosome occupancy as a novel feature in our model. 4) We have used seven diverse species to validate the generality of our four-feature model, a far wider range of species than has been attempted when validating existing methods. The seven species are *Homo sapiens *(human), *Mus musculus *(mouse), *Gallus gallus *(chicken), *Caenorhabditis elegans *(*C.elegans*), *Oryza sativa *(rice), *Arabidopsis thaliana *(Arabidopsis), and *Solanum lycopersicum *(tomato). Intriguingly, the performance of our model on cross species predictions reflected the phylogenetic distances among these seven eukaryotes.

## Materials and methods

### Notation of region

We use the notation < ± N, ± M > to denote a region with respect to the cleavage site, where N and M are the number of nucleotides (nts) upstream ('-') or downstream ('+'), from the cleavage site.

### Poly(A) site discovery

Poly(A) sites were discovered by mapping polyadenylated ESTs and/or cDNAs to the reference genomes as described in [15]. Briefly, our method involves: a) ESTs that either terminate with at least eight polymeric 'A' at the 3' end, or start with at least eight polymeric 'T' at the 5' end are selected. For cDNA datasets, only sequences that terminate with at least eight 'A' are selected. b) Polyadenylated ESTs and cDNAs are mapped to the appropriate reference genome using NCBI BLAST 2.2.23. C) Customized Python scripts are used to determine the direction of transcription, and to eliminate artifacts due to false oligo-dT priming.

ESTs and cDNAs sequences were downloaded from NCBI's dbEST, and Refseq databases. Through this method, 22,479, 8,779, 1,292, 845, and 6,380 poly(A) sequences were discovered in human, mouse, chicken, *C.elegans*, and tomato, respectively. Genomes of human Build 7, mouse Build 9, chicken May 2006 release, *C.elegans *Feb 2000 release, and tomato Nov 2010 release 2.31 were used for mapping.

### Other poly(A) sequences

We used reliable published datasets of Arabidopsis poly(A) sequences from http://www.users.muohio.edu/liq/Loke_et_al_2005_8k[20] and rice from http://www.users.muohio.edu/liq/Rice_55K_PolyA_site_dataset.zip[21]. We eliminated poly(A) sequences that could not be mapped to the reference genomes. Arabidopsis genome Build 9 2009 and rice genome Build 4.0 Jun 2010 were used. As a result, we compiled 8,160 and 41,046 poly(A) sequences from Arabidopsis and rice, respectively.

### Transcribed non-poly(A) sequences

For specificity testing, 1,000 spliced and unspliced transcribed sequences were chosen randomly from Arabidopsis and human datasets. Gene sequences of Arabidopsis were downloaded from ftp://ftp.arabidopsis.org/home/tair/Sequences/blast_datasets/TAIR10_blastsets/, whereas human sequences were prepared by customized Python programs. For sequences longer than 600 nts, 600-nt long fragments were extracted at random locations.

### Position-by-kmer matrix

In order to examine the localization of kmers (oligonucleotides of length k) in a set of sequences, we captured and converted sequence information into a matrix so that it could be processed by PCA (see below, and Results and discussion). Starting from the leftmost position of a sequence, we use a sliding window of size k to count the occurrence of a kmer and its position. The occurrence of each kmer at various positions is stored in the column of the matrix, so that each row stores the counts of all possible kmers (4^k^) at a position. A row is a 4^k ^-dimensional vector, where each dimension is the occurrence of a kmer at a position. Each column is the occurrences of a kmer at different positions.

### Position score matrix (PSM)

Position score matrix (PSM) is a M-by-N matrix, where M is equal to 4^k^, *k *is the size of oligonucleotide. And, N is (*l*-*k*+1), where *l *is the length of considered region. For our model construction, we consider region < -100, +100 >, i.e. *l *is equal to 200. The value of each entry in PSM, *v(kmer, n)*, is defined as below:

$$v\left[kmer,n\right]={\text{log}}_{2}\left(\frac{observed_{real}kmer\text{\_}at\text{\_}position\text{\_}n}{observed_{false}kmer\text{\_}at\text{\_}position\text{\_}n}\right)$$

The value v(kmer, n) represents the fold difference of observing a particular kmer at position *n *in real versus false poly(A) sequences, where *n *is in the range of 1 to (*l*-*k*+1). Given a sequence, a k-sized window is used to slide through it one position at a time from left to right. The result is a list of overlapping kmers with their corresponding starting positions in the sequence (a list of (kmer, n) pairs). This list will be used to retrieve the associated values from PSM i.e. v(kmer, n), and the sum of these values is the PSM score of the sequence.

### Nucleosome occupancy matrix (NOM)

NOM is constructed by a similar method used in building the PSM. The nucleosome prediction method developed by Segal lab [22] was used to determine the probability of nucleosome occupancy in each position in < -500, +500 > for real and false poly(A) sequences. Due to boundary effects, predictions from 200 nts at both ends are discarded, leaving only the middle < -300, +300 > region for consideration. Like PSM, the value of each entry in the NOM, v(*p*_n_), is a defined as below

$$v\left[p_{n}\right]={\text{log}}_{2}\left(\frac{occupancy\text{\_}prob\text{\_}at\text{\_}position\text{\_}n_{real}}{occupancy\text{\_}prob\text{\_}at\text{\_}position\text{\_}n_{false}}\right)$$

*p*_n _is the predicted occupancy probability at position *n*.

### Feature vector

The feature vector consists of four features: trimer PSM scores for < -100, -1 > and < +1, +50 >, dimer PSM score across the cleavage site < -10, +10 >, and the nucleosome occupancy score based on NOM in the region < -300, +300 >. Feature vectors are standardized by the mean and standard deviation obtained from all real and false poly(A) sequences within each species' dataset.

### Performance measurement

Prediction is measured by sensitivity (Sn), specificity (Sp), and Matthews correlation coefficient (MCC)[23]. Sn = Tp/(Tp+Fn), Sp = Tn/(Tn+Fp), MCC = (Tp•Tn-Fp•Fn)/√(Tp+Fp)•(Tp+Fn)•(Tn+Fp)•(Tn+Fn), where Tp, Tn, Fp, and Fn denote true positive, true negative, false positive, and false negative, respectively.

### Training and testing procedures

False poly(A) sequences (negative dataset) were generated according to the 2^nd ^order Markov model obtained from real poly(A) sequences in region < -300, +300 >. Accuracy was reported as the average of twenty ten-fold cross validations. Linear discriminant analysis (LDA) and logistic regression (LR) were performed by Python machine learning package mlpy [24].

### Method comparisons

Polya_svm [25] version 2.1 was downloaded from http://exon.umdnj.edu/polya_svm/, and PolyA-EP [16] was downloaded from http://mlkd.csd.auth.gr/PolyA/tools.html. Polya_svm was tested on 22,479 human poly(A) sequences, each consisted of 100 nts upstream and downstream from the poly(A) site. For PolyA-EP, 8,160 Arabidopsis poly(A) sequences were used where each consisted of the 300 nts upstream and 100 nts downstream regions, relative to cleavage sites.

### 18S, GAPDH, and CPSF3 sequences, and phylogenetic distance calculations

The evolutionary relationship among these seven eukaryotes was determined through phylogenetic analysis of three independent gene products: the ribosomal 18S, gylceraldehyde 3 phosphate dehydrogenase (GAPDH) and cleavage and polyadenylation specificity factor subunit 3 (CPSF3). 18S genes of human (NR_003286), mouse (NR_003278), *C. elegans *(EU196001), *O. sativa *(AF069218), *S. lycopersicum *(X51576), and *A. thaliana *(AT2G01010) were obtained from NCBI. Chicken's 18S was determined by a BLAT search [26] using human's 18S as query sequence in UCSC genome browser, http://genome.ucsc.edu[27]. Homologous GAPDH protein sequences for all organisms except *S. lycopersicum *were obtained from the Homologene database in NCBI (the Homologene ID of GAPDH gene is 107053). The GAPDH sequence of *S. lycopersicum *was obtained by translating its cDNA sequence AK322678. Similarly, homologous CPSF3 protein sequences (ID 6499) were collected from the Homologene database, except for *S.lycopersicum*, where the CPSF3 was obtained by the translation of AK327795. Sequences were aligned by T_COFFEE [28]. Phylogenetic distances between the seven species were calculated by dnadist (18S) and protdist (GAPDH, CPSF3) in PHYLIP [29].

## Results and discussion

### Feature identification by Principal Component Analysis (PCA)

**Figure 1:**
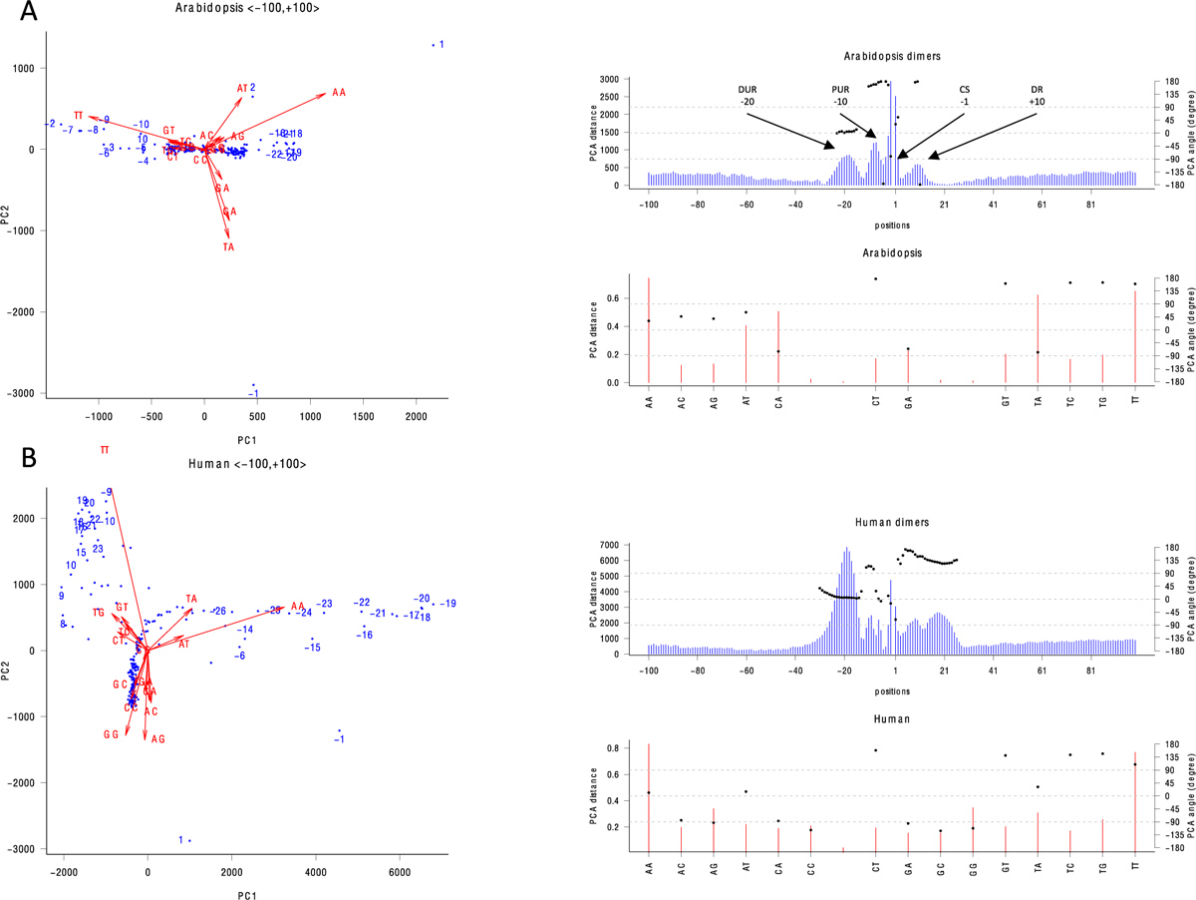
**Dimer PCA of real poly(A) sequences**. Blue dots represent positions, and red arrows represent kmers. Only arrows longer than a specified threshold are labeled. DUR, PUR, CS, and DR denote distal upstream region, proximal upstream region, cleavage site, and downstream region, respectively. A) Arabidopsis, B) human.

Simulated sequences were used to prove the viability of our PCA method (see Additional file 1), which we then applied to Arabidopsis and human data using different sizes of kmers. Figure 1A shows PCA of Arabidopsis poly(A) sequences using dimers. In the PCA-position profile (top right panel of Figure 1A), four localized groups (peaks) are identified: -20 (distal upstream region, DUR), -8 (proximal upstream region, PUR), -1 (cleavage site, CS), and +10 (downstream region, DR). Examining the PCA-oligo profile (bottom right of Figure 1A), 'AA' and 'AT' are most often found in the DUR. 'TT' is the most significant dimer in the PUR and DR. Note that the extent of localization in DR is moderate versus far upstream and downstream regions. This result concurs with current view that poly(A) sites of plants are mainly characterized by upstream elements [30]. 'CA', 'GA', and 'TA' (-90 degrees) are found in CS, and of these, 'CA' is predominant. Similar to Arabidopsis, human (Figure 1B) PUR is also found to localize in four regions, but with different relative concentration of dimers. DUR at -20 shows the strongest localization of 'AA', and then followed by 'AT' and 'TA'. PUR at -10 shows a weak localization (compared to DUR) of 'TT'. The human CS at +1 shows weaker localization compared with Arabidopsis' CS. However, seven dimers ('AC', 'AG', 'CA', 'CC', 'GA', 'GC', and 'GG') are likely to localize at the human CS (-90 degrees). The Human DR covers an extensive region from +1 to +23, which is absent in Arabidopsis. 'CT', 'GT', 'TC', 'TG', and 'TT' are the main dimers in human DR.

Even with trimer motifs, there are no prominent cis-elements in the downstream region of Arabidopsis poly(A) sites. The downstream elements (DSE) are not essential for polyadenylation in the invertebrate *C.elegans *[31]. These results might indicate that DSE, which are essential in vertebrates, evolved more recently than other poly(A) site elements. Moreover, we do not find core poly(A) cis-elements stretching beyond 40 nts upstream from the cleavage site in Arabidopsis, which is at odds with an early bioinformatics study [32,33]. Certain genes do possess specific cis-elements in a much further upstream region [15,34,35], but there weren't sufficient signals across all genes for this to be a key feature of poly(A) sites. In order to construct a model that can embrace such a diversity in the poly(A) sites in different species, the features obtained from the region flanking the poly(A) site are split into three subregions approximately according to the peaks as shown in Figure 1, i.e. < -100, -1 >, < -10, +10 >, and < +1, +50 >. The purpose of doing that is to prevent the contributing effect of features from one subregion to be cancelled by non-contributing features from other subregions.

Nevertheless, poly(A) sites may be constituted of longer sequence structures, and we continued PCA for longer kmers. Differences between dimer and trimer profiles (Figure S2A in Additional file 1) are insignificant in both Arabidopsis and humans. However, when the size of kmers is increased to six, the localization signal at the CS drops or even vanishes in both species (PCA-position profiles in Figure S2A-B), meaning that the size of elements favored at the CS is shorter than 6 nts, and may be identified well by dimers or trimers. With hexamers, the significant signal in the human PUR vanishes (right of Figure S2B) but not for Arabidopsis. The DR of human becomes flat relative to the DUR and its signal disappears entirely in the octamer PCA-position profile. Hence, the size of sequence elements in the human DR should be 3-5 nts. In both species, the canonical poly(A) signal AAUAAA has the longest PCA-distance according to the PCA-oligo profiles in Figure S2B. Interestingly, unlike humans, the DUR and PUR of Arabidopsis still remain even in octamer PCA-position profile (Figure S2C). As the poly(A) signal is highly conserved, the hexamer profiles presented above may lead one to believe that the poly(A) signal and/or hexamers are effective features for poly(A) site recognition.

On the contrary, our study suggests the opposite (see Additional file 1 for a test of hexamer vs trimer features). The Matthews correlation coefficient (MCC) attained by the trimer model was 0.76 versus 0.73 for the hexamer model, indicating the trimer model had more accurate predictive power. Furthermore, we compared the false positive rates of predicting false poly(A) sequences between the trimer model and an existing method, polya_svm [25], which uses a hexamer as an upstream feature. Surprisingly, the false positive rate for the hexamer feature-containing polya_svm was 82%, while that of the trimer model was just 17%. Clearly, hexamer models do not offer substantial advantages relative to a trimer model.

Additionally, the model with hexamer features demands a larger training sample (> 4,000 sequences) than the trimer model because smaller samples are unlikely to capture the full distribution of all genomic contexts for poly(A) hexamers within a species. Complicating hexamer model training is the fact that poly(A) signallike hexamers are ubiquitous in large genomes in places that are not poly(A) sites, such as introns. Eukaryotic cells likely enhance the recognition and regulation of the actual poly(A) sites with other auxiliary cis-elements that may be shorter than hexamers [36], which could be better distinguished with a model looking for smaller features. This viewpoint is further supported by the fact that intra-species poly(A) sites are highly variable and yet they are recognized by the same polyadenylation apparatus. Thus, we choose trimers instead of hexamers to form part of the feature vector.

### Nucleosome structure at poly(A) sites

**Figure 2:**
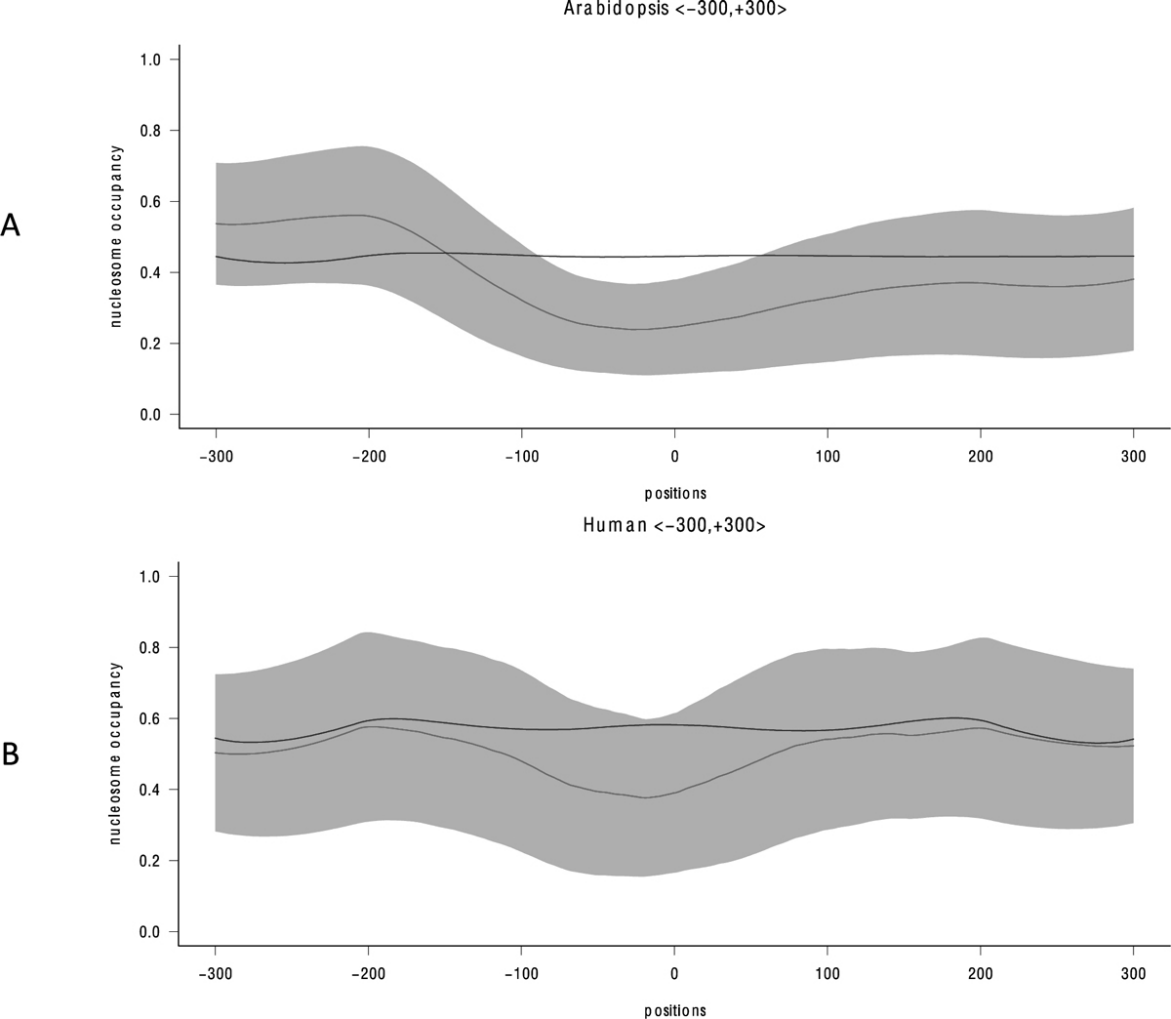
**Predicted nucleosome occupancy in region 300 nts upstream and downstream of poly(A) sites**. The vertical axis is predicted probability. Red and blue lines represent average probability of occupancy for real poly(A) sites, and 2^nd ^order Markov sequences i.e. false sequences, respectively. The shaded region denotes the 95% confidence interval. A) Arabidopsis, B) human.

Previous studies suggested that nucleosomes are depleted in the proximity of poly(A) sites [19,37,38], but no poly(A) site prediction models have yet incorporated this feature. Hence we utilize a nucleosome occupancy prediction method developed by the Segal lab [22] to explore nucleosome occupancy in the region flanking the poly(A) sites (Figure 2). Even though the false poly(A) sequences mimic the 2^nd ^order Markov property of real poly(A) sequences, their predicted nucleosome occupancy remains at a steady level throughout the entire region. In contrast, both human and Arabidopsis actual poly(A) sequences show a reduced likelihood of nucleosomes around the cleavage site. Our results indicate a putative relationship between nucleosome depletion and polyadenylation site, in agreement with published works [19,37,38]. We have thus included predicted occupancy in the feature vector. The role of nucleosome formation on polyadenylation is currently unknown, and should be further investigated. We speculate nucleosome formation may influence the selection of alternative poly(A) sites in genes, as 54%, 32% and 70% of human, mouse, and Arabidopsis genes, respectively, contain multiple poly(A) sites [14,39].

### Feature vector and poly(A) site predictions

After a thorough analysis of poly(A) sequences, we have selected four features in the feature vector to signify a poly(A) site (upstream trimers, downstream trimers, dimers near to the cleavage site and the nucleosome occupancy, see Materials and methods). We validated the efficacy of these features by two independent supervised machine learning (ML) methods: logistic regression (LR) and linear discriminant analysis (LDA). LDA requires a Gaussian distribution of feature values but LR does not. Quadratic discriminant analysis (QDA) was also considered since the positive and negative datasets might exhibit different covariance, but we did not find any significant differences between LDA and QDA in terms of prediction accuracy (data not shown). For simplicity, we report our results using LDA. The optimal performance peaks when thresholds are between 0.5 and 0.6 according to the receiver operating characteristic (ROC) method (Figure S3 in Additional file 1), therefore the threshold for LR is set to 0.5 for the rest of the analysis. The two ML methods were tested using poly(A) sequences from seven species including two mammals, a bird, an invertebrate, two dicots, and a monocot. Our two ML methods achieved 85-92% specificity and 85-96% sensitivity for each of the seven species (Table 1). Results from the logistic and LDA show no significant differences (data not shown). Since no existing method has been used for multispecies predictions, we must compare our methods to other methods on individual species.

**Table 1:** Multispecies poly(A) signal predictions are assessed by sensitivity (Sn), specificity (Sp), and Matthews correlation coefficient (MCC) in seven diverse species.

	LDA			LR		
	
	Sn	Sp	MCC	Sn	Sp	MCC
Human	85	93	0.78	86	90	0.77
Mouse	91	96	0.87	92	94	0.86
Chicken	87	91	0.78	87	90	0.77
C.elegans	85	89	0.74	90	85	0.75
Oryza sativa	88	89	0.77	88	89	0.77
Arabidopsis	92	92	0.84	91	91	0.82
s.lycopersicum	91	92	0.83	90	91	0.81

Sn and Sp are expressed in percentage.

### Methods comparison and model validation

We compared our LR and LDA models to two poly(A) site prediction methods, polya_svm [25], and PolyA-EP [16]. The former was originally developed for animals and the latter was for plants. Both of our simpler, four-feature LDA and LR models produced better results than polya_svm in predicting human poly(A) sites in terms of sensitivity and specificity (Table 2). The Polya-EP model is skewed towards sensitivity when analyzing Arabidopsis data, while our models performed well and without bias. As our models were trained on the Arabidopsis dataset built by the PAC method [20], we can also compare our two ML methods with this third published method without repeating the tests. The best MCC achieved by PAC is between 0.65 to 0.70 with various types of negative samples and positions of deviation (Figure 3 of [20]). But our two ML methods are able to improve MCC significantly to 0.82-0.84 (Table 2). Additionally, we conducted three more tests to validate our model: i) prediction for other transcribed, non-poly(A) genomic sequences, ii) testing the relative contribution of each feature to accuracy, and iii) prediction of larger poly(A) regions. These results can be found in the Additional file 1.

**Table 2:** Comparing LDA and LR methods with polya_svm and PolyA-EP.

		Sn	Sp	MCC
Human	LR	86	90	0.77
	LDA	85	93	0.78
	Polya_svm	84	89	0.73

Arabidopsis	LR	91	91	0.82
	LDA	92	92	0.84
	PolyA-EP	95	41	0.43
	PAC	-	-	0.65-0.70

Sn and Sp are expressed in percentages. The MCC of PAC is from Figure 3 of [20].

### Cross species poly(A) site predictions

As polyadenylation is a universal function across the tree of life, we are interested in the evolution of poly(A) sites across diverse species. However, because poly(A) sites lie in relatively unconstrained untranslated regions (3'UTR), aligning these sequences is usually infeasible. As a result, prevalent phylogenetic methods are unsuitable to look at the evolution of poly(A) sites. Our method, applied across species, is able to recapitulate the phylogenetic relationship across species based on non-coding sequences. The phylogenetic distances among these seven organisms (Table 3) was established through phylogenetic trees, built independently using three unlinked genes (Figure S5A-C left panel in Additional file 1). All three phylogenetic trees not only produced congruent topology, but also similar evolutionary distances (correlations between gene products > 0.75, p-value < 10^-10^).

**Table 3:** Phylogenetic distances between species based on, A) 18S, B) GAPDH protein, C) CPSF3.

Species1	Species2	18S	GAPDH	CPSF3	rSn
mouse	human	0.008099	0.083105	0.01452	88.5
chicken	human	0.034382	0.080086	0.052188	85.0
c.elegans	human	0.336445	0.309192	0.642712	68.5
O.sativa	human	0.245416	0.3565	0.674258	61.0
Arabidopsis	human	0.240724	0.3788	0.678983	65.5
S.lycopersicum	human	0.232462	0.390498	0.838763	58.0
chicken	mouse	0.035031	0.098663	0.061379	86.0
c.elegans	mouse	0.336259	0.318807	0.649077	67.0
O.sativa	mouse	0.242306	0.376424	0.677389	60.5
Arabidopsis	mouse	0.24307	0.40044	0.683073	55.5
S.lycopersicum	mouse	0.233802	0.390069	0.843015	58.0
c.elegans	chicken	0.331649	0.26794	0.63646	67.5
O.sativa	chicken	0.242221	0.338926	0.688556	61.5
Arabidopsis	chicken	0.241121	0.353368	0.685943	62.0
S.lycopersicum	chicken	0.231997	0.36337	0.843798	54.0
O.sativa	c.elegans	0.392745	0.388677	0.848847	65.0
Arabidopsis	c.elegans	0.3879	0.40471	0.885896	70.5
S.lycopersicum	c.elegans	0.377503	0.387265	0.997973	64.0
Arabidopsis	O.sativa	0.054837	0.260299	0.245492	63.5
S.lycopersicum	O.sativa	0.045931	0.273969	0.346818	68.5
S.lycopersicum	Arabidopsis	0.031789	0.218126	0.31507	65.5

Unit is substitution per site. The last column rSn is defined according to Materials and methods section.

We utilize the reciprocal sensitivity (rSn) (see Materials and methods) to assess the overall homology of poly(A) sites between species. Sensitivity was obtained by applying the logistic model of one species to predict real poly(A) site sequences of another species (Table 4). The validity of the poly(A)-based phylogenetic relationship is measured by the correlation between gene-based phylogenetic distances and rSn predicted by the logistic model. The correlation coefficients are -0.50, -0.87, and -0.77 based on the 18S, GAPDH, and CPSF3 trees, respectively (p-value < 10^-5^) (Figure S5A-C in Additional file 1). Given the high intraspecies variability, nearly no two poly(A) sites are alike in the same genome [15], there should therefore not necessarily be conservation in poly(A) sites between more closely related species. However, our results show that prediction accuracy decreases when the model from one species is applied to data from other species and the decrease is proportional to phylogenetic distance.

**Table 4:** Cross species predictions measured by sensitivity.

	Poly(A) sites						
Models	Human	Mouse	Chicken	C.elegans	Oryza sativa	Arabidopsis	S.lycopersicum
Human	**87**	**90**	**90**	**73**	58	86	90
Mouse	**87**	**93**	**90**	**72**	60	75	90
Chicken	**80**	**82**	**96**	**71**	53	78	81
C.elegans	**64**	**62**	**64**	**95**	60	85	83
Oryza sativa	64	61	70	70	**89**	**93**	**98**
Arabidopsis	45	36	46	56	**34**	**94**	**79**
s.lycopersicum	26	26	27	45	**39**	**52**	**93**

Sensitivity is calculated by using model from one species (indicated in the leftmost column) to predict real and false poly(A) sequences from other species (top row). The table needs not be symmetrical because poly(A) sites from different species tend to possess different characteristics according to the PCA profiles discussed above.

This leads us to question about the boundary of potential poly(A) sites, which we can conceptualize as PA-space, the allowable variations of functional poly(A) sites. Core polyadenylation proteins that directly bind to the pre-mRNA, such as CPSF1 and CstF-64, are likely to co-evolve with the PA-space. While some studies have identified conserved residues in CstF-64 that correlated with poly(A) downstream elements [33], no one has looked at how variation in the nucleotide sequence and proteins are correlated and coevolving. Currently, the sequence-level dynamics between RNA binding proteins and their substrates is unclear, but the decreasing costs of sequencing transcriptomes should provide data from a wider range of species soon.

## Conclusions

We have shown the feasibility of conducing comprehensive genomic analysis of poly(A) sites using PCA, a method which could be broadly applied for any cis-element identification. We believe a model focused on very short oligonucleotides outperformed those with hexamers as features because it embraces the conspicuous poly(A) signal elements without sacrificing the diverse family of auxiliary biological signals surrounding the poly(A) sites. We also included for the first time nucleosome occupancy as an informative predictor of poly(A) sites.

## List of abbreviations

3'UTR: 3' untranslated region; CS: cleavage site; DR: downstream region; DSE: downstream element; DUR: distal upstream region; FN: false negative; FP: false positiv;. LDA: linear discriminant analysis; LR: logistic regression; MCC: Matthews correlation coefficien;. ML: machine learning; NOM: nucleosome occupancy matrix; PCA: principal component analysis; poly(A): polyadenylation; PSM: position score matrix; PUR: proximal upstream regio; QDA: quadratic discriminant analysis; ROC: receiver operating curve; rSn: reciprocal sensitivity; Sn: sensitivity; Sp: specificity; TN: true negative; TP: true positive.

## Competing interests

The authors declare that they have no competing interests.

## Authors' contributions

ESH conceived of the study, designed and carried out the analyses and drafted the manuscript. SIG conceived of the study. SD conceived of the plant portion of the study, assisted in data analysis and helped draft the manuscript.

## Supplementary Material

Additional file 1 **Table S1: Existing poly(A) sites methods**. Figure S1: Feature identification by Principal Component Analysis (PCA). Figure S2: PCA of real poly(A) sequences. The canonical poly(A) signal is a misguiding feature. Figure S3: ROC of logistic method. Table S2: False positive rate (in percentage) committed by different methods in handling CDS gene sequences. Table S3: Relative contribution of individual features. Figure S4: Predictions of five hundred 2,000-nt poly(A) sites by sliding a 600-nt sliding window from left to right. Figure S5. Correlation between phylogenetic distance and reciprocal sensitivity (rSn) between seven species: human, mouse, chicken, c.elegans, rice, Arabidopsis, and tomato. Click here for file

## Declarations

The publication fee of this article is funded by Rutgers School of Environmental and Biological Sciences and the New Jersey Agricultural Experiment Station (NJAES). This work is supported by National Institute of Health (NIH) grant NIH K12 GM093854-01 to E.S.H. S.I.G. is supported by NIH GM57286. S.D. is supported by funds from the NJAES.

This article has been published as part of *BMC Bioinformatics *Volume 14 Supplement 2, 2013: Selected articles from the Eleventh Asia Pacific Bioinformatics Conference (APBC 2013): Bioinformatics. The full contents of the supplement are available online at http://www.biomedcentral.com/bmcbioinformatics/supplements/14/S2.
