# Supplementary material for: A multispecies polyadenylation site model
Source: BMC Bioinformatics. 2013 Jan 21;14(Suppl 2):S9. doi: 10.1186/1471-2105-14-S2-S9 (PMC3549828; doi:10.1186/1471-2105-14-S2-S9)
Supplement: Additional file 1 — Table S1: Existing poly(A) sites methods. Figure S1: Feature identification by Principal Component Analysis (PCA). Figure S2: PCA of real poly(A) sequences. The canonical poly(A) signal is a misguiding feature. Figure S3: ROC of logistic method. Table S2: False positive rate (in percentage) committed by different methods in handling CDS gene sequences. Table S3: Relative contribution of individual features. Figure S4: Predictions of five hundred 2,000-nt poly(A) sites by sliding a 600-nt sliding window from left to right. Figure S5. Correlation between phylogenetic distance and reciprocal sensitivity (rSn) between seven species: human, mouse, chicken, c.elegans, rice, Arabidopsis, and tomato. [file 1471-2105-14-S2-S9-S1.pdf]

ADDITIONAL FILE 1

A multispecies polyadenylation site model

Eric S. Ho[1,*], Samuel I. Gunderson[2], Siobain Duffy[3]

1. Department of Molecular Genetics, Microbiology and Immunology, University of Medicine and Dentistry of New Jersey- Robert Wood Johnson Medical School, Piscataway, New Jersey, USA

2. Department of Molecular Biology and Biochemistry, Rutgers University, New Brunswick, New Jersey, USA

3. Department of Ecology, Evolution and Natural Resources, Rutgers University, New Brunswick, New Jersey, USA

*Corresponding author: eric.ho@umdnj.edu

Existing poly(A) sites methods

Table S1: A sample of nine existing methods, number of features used by the models, and organism data. DSM/HMM and Erpin are built on hidden Markov model.

Method Name	Number of features	Organism Data	References
Polyadq	6	human	[1]
DSM/HMM	N.A.	*S.cerevisiae*	[2]
Erpin	N.A.	human	[3]
LIU-SVM*	113	human	[4]
Polya_svm	15	human	[5]
SMO*	228	Arabidopsis	[6]
polyA-EP	5460	Arabidopsis	[7]
PAC	21	Arabidopsis, rice	[8]
DPS	274	human	[9]

*Names assigned by authors as no name were given in the original papers.

Feature identification by Principal Component Analysis (PCA)

For a proof of concept, we simulated 2,000 200-nt long sequences where all types of nucleotides were equiprobable. Sequence positions were labeled from -100 to +100 to

order to mimic poly(A) sequences where the cleavage site is at the midpoint. We setup an uneven distribution of dinucleotides (“dimers”) by tagging these sequences with designated dimers as follows:

a) ‘AC’ was specially planted at -90 and +90 in each sequence
b) Half of the sequences at +12 were tagged with ‘GT’, and the other half were tagged with ‘AT’
c) ‘CG’ was planted at -10 in half of the sequences
d) ‘TA’ was planted at +52 in each sequence

These sequences were first transformed into a position-by-2mer matrix (200 rows and 16 columns) before being processed by PCA using R [10] (Figure S1A). Description of position-by-kmer matrix can be found in Materials and Methods section. Each blue dot corresponds to a position in the sequence. Its distance from the origin reflects the distribution of dimers at that position. If the distribution is skewed, implying localization, the data point will reside far from the origin. Arrows in red represent individual dimers. Their lengths reflect the extent of localization of the dimers they represent. The longer the arrow, the higher is the localization. Blue dots pointed by the arrow indicate the most frequent locations of the dimer. The angle between arrows reflects the correlation between the pair of dimers. The smaller the angle, the higher is the correlation. Note that it is inappropriate to compare the length of an arrow with the distance of a position (blue dot) from the origin. Only positions and arrows show unusual localization are labeled (Figure S1A). Localizations of planted dimers ‘AC’ and ‘CG’ in respective positions are noticeable. However, it is less obvious for ‘CG, ‘GT’ and ‘AT’ due to their moderate localizations, which is reflected by the length of arrows. One issue faced by the typical PCA plot (Figure S1A) is that it turns crowded when too many arrows and/or positions are considered in the case of longer kmer model and/or larger region. An alternative is to view the distance of positions (blue dots in Figure S1A) and kmers (red arrows in Figure S1A) from the origin, namely PCA-distances, as shown Figure S1C. We call Figure S1C PCA-position profile. Each blue vertical bar denotes the PCA-distance (units in left y-axis) of each position from the origin. Black dots represent the direction pointed by corresponding positions. Direction is measured by the angle θ, called PCA-angle, as

diagrammed in Figure S1B. Note that angles near to +180 and -180 should be considered to point to the similar direction. PCA-angle is measured degrees according to the y-axis on the right. The same idea is applied to display the PCA-distance of dimers as shown in Figure S1D, namely PCA-oligo profile. Black dots indicate the direction pointed by the corresponding dimers (red arrows) in Figure S1A. The five sharp peaks reveal certain dimers are localized in those positions (Figure S1C). As shown in the PCA plot (Figure S1A), these positions and dimers point approximately to same direction. Thus, the common PCA-angle shared between positions in PCA-position profile and kmers in PCA-oligo profile serves as the key to identify the localized kmers in the sequences. For example, the peak in position +12 points to ~135 degree (Figure S1C), which corresponds to ‘AT’, ‘GT’ and ‘TT’ in Figure S1D. ‘TT’ likely overlaps with ‘GT’ in 3’ direction as its PCA-distance is slightly shorter. Hence, simulation results support the use of PCA to unravel hidden localizations in the sequences.

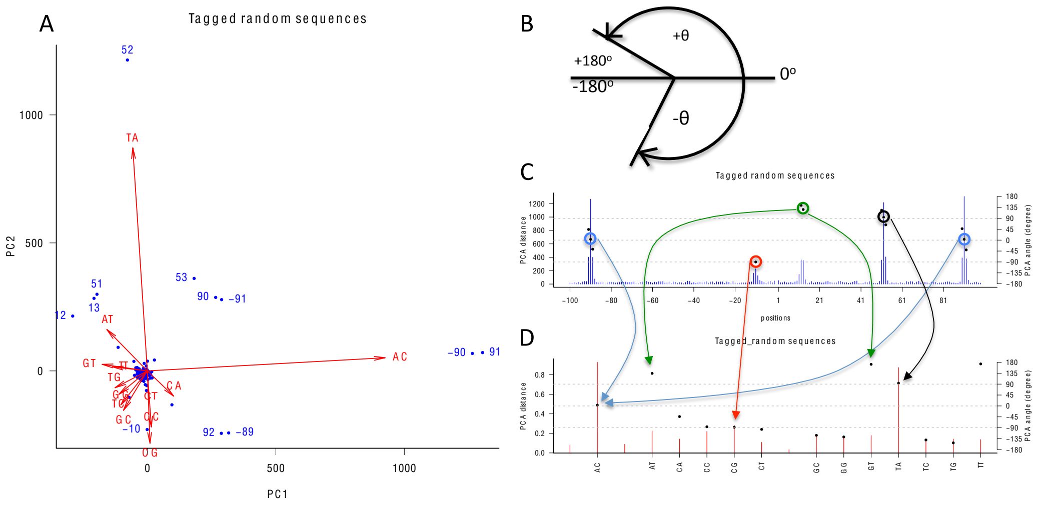

Figure S1: Feature identification by Principal Component Analysis (PCA) of simulated sequences. Blue dots, and red arrows represent positions, and kmers, respectively. A) PCA plot of simulated sequences. Only outlying positions and kmers are highlighted. B) definition of angle θ. Anti-clockwise is positive, clockwise is negative. C) PCA-position profile. D) PCA-oligo profile. Color coded arrows connect the positions and kmers sharing the same PCA-angle.

PCA of Arabidopsis and Human for trimers, hexamers and octamers

A

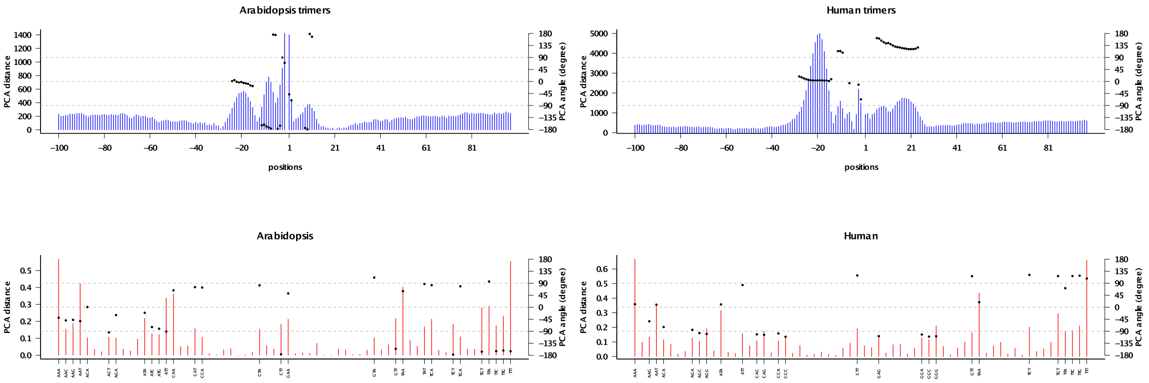

B

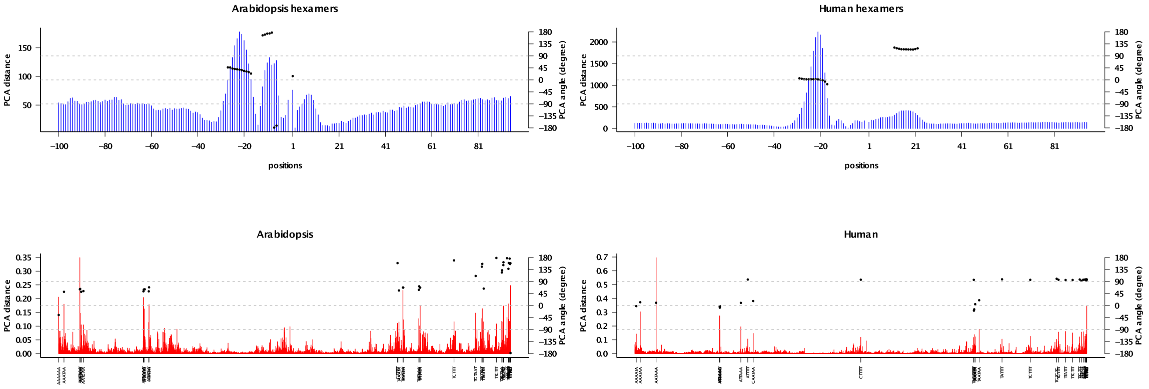

C

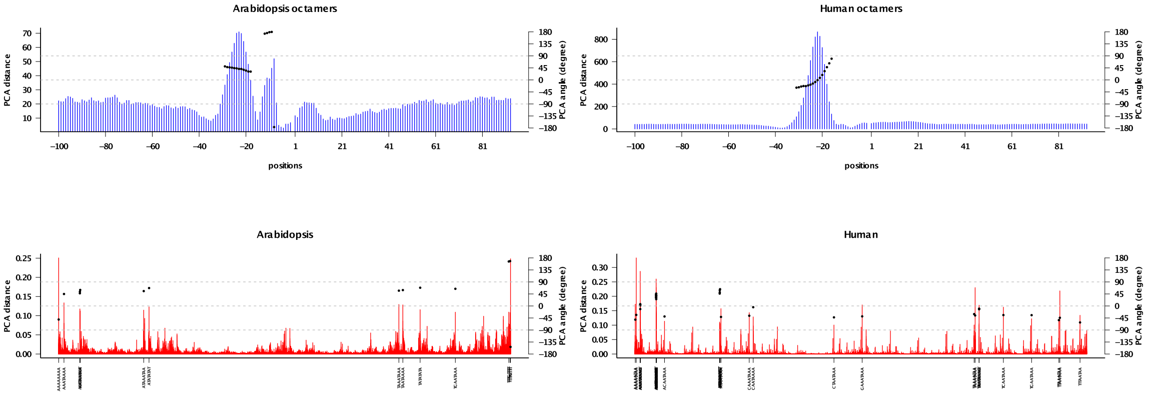

Figure S2: PCA of real poly(A) sequences. Blue dots, and red arrows represent positions, and kmers, respectively. Only arrows longer than specified threshold are labeled. A) Trimer , B) hexamer, C) octamer.

The canonical poly(A) signal is a misguiding feature

To test this, two sets of false poly(A) sequences were used. One set composes of 2nd order Markov simulated sequences tagged with the AATAAA at random positions in the region <-30,-10>. The other set contains 1,207 human intergenic sequences with AATAAA in the same upstream region. In both categories, the trimer model produces fewer false positive errors than the hexamer model. For simulated dataset, false positive rates committed by the trimer and hexamer models were 17% and 20%, respectively. With the human intergenic sequences, false positive rates are 57% and 84% for the trimer and hexamer models, respectively. The hexamer models acheived 88% sensitivity in predicting human poly(A) sequences, which was 3% higher than the trimer model. However, the trimer model attained 90% specificity compared to 83% in the hexamer model.

In addition, polya_svm [5] was used to make predictions for the simulated sequences. It committed 82% false positive rate.

Receiver operating characteristic (ROC)

ROC method was used to determine the optimal threshold for predictions produced by the logistic model (Figure S3). The ROC plot illustrates the influence of threshold on true positive rate (sensitivity) and false positive rate (specificity) for Arabidopsis and human models. According to ROC, the model achieved optimal performance when threshold was set between 0.5 and 0.6. Thus, the threshold for logistic method is set to 0.5 such that a sequence scored above 0.5 is considered as a poly(A) site; negative if otherwise.

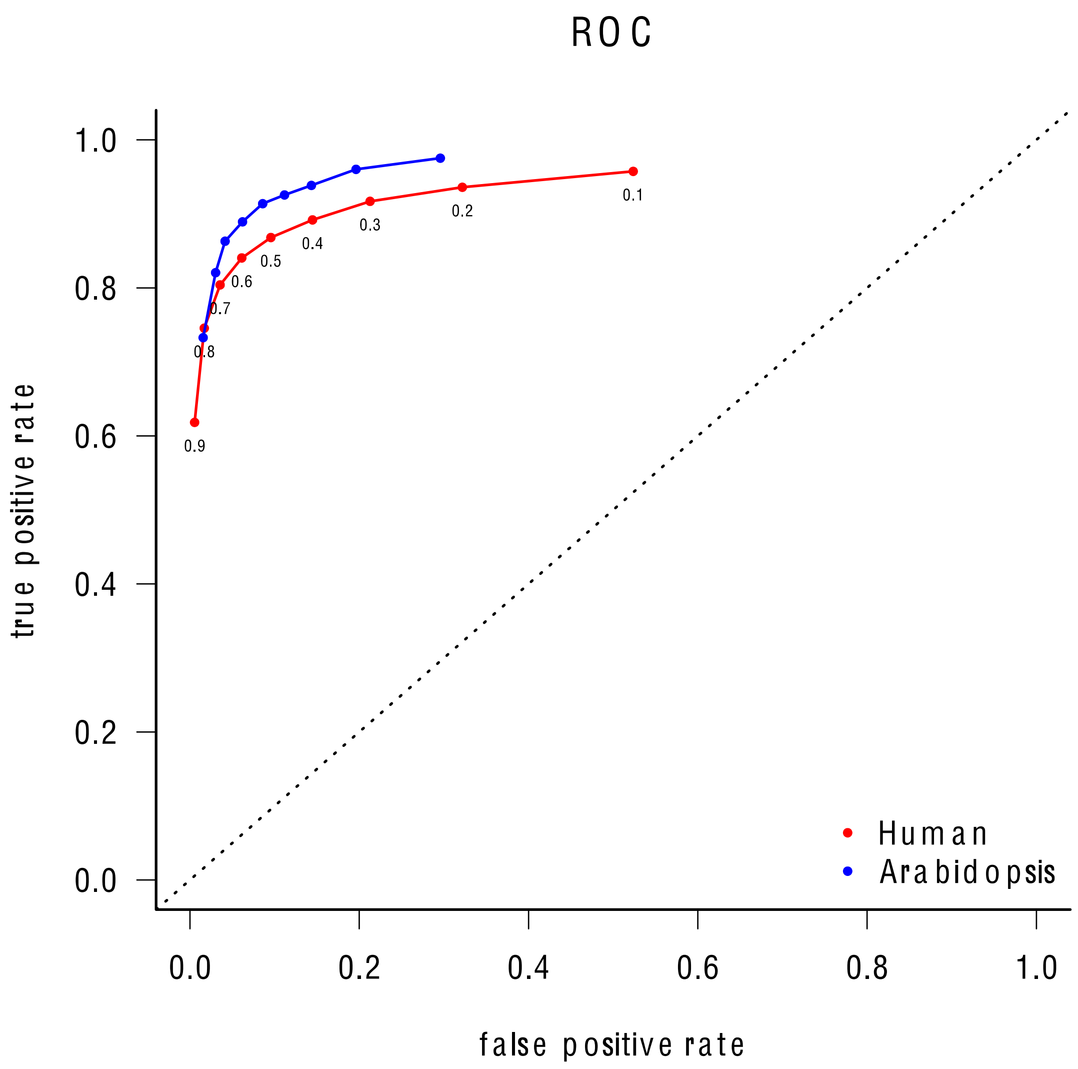

Figure S3: ROC of logistic method. Thresholds vary from 0.1 to 0.9 with increment of 0.1. Arabidopsis, and human predictions are colored in red, and blue, respectively.

Predictions in transcribed, non-poly(A) sequences

Besides poly(A) sites, we have also considered how well our methods can differentiate poly(A) sequences from other transcribed, non-poly(A) genomic sequences. Two types of sequences were used to test this point. One is the coding sequence (CDS) in which introns are spliced out. The other is the unspliced gene sequence. As shown in Table S2 below, our methods perform better than the other two methods in both types of transcribed sequences in human and Arabidopsis.

Table S2: False positive rate (in percentage) committed by different methods in handling CDS gene sequences.

		CDS	Gene
Human	LR	5	17
	LDA	4.5	15
	Polya_svm	11	19
Arabidopsis	LR	2.3	3.3
	LDA	2	3.1
	PolyA-EP	2.5	7.1

Relative contribution of features

We determined the contribution of each feature in prediction. We did that by using only a single feature each time to build the model, and then assessed its performance relative to the full model (Table S3). In humans, the features in decreasing importance are the upstream trimer, the cleavage dimer, the downstream trimer, and the nucleosome occupancy. Such order persists regardless of the ML methods. The relative contribution of features for Arabidopsis shows that the importance of the least important features are reversed compared to humans: the upstream trimer, the cleavage dimer, the nucleosome occupancy, followed by the downstream trimer. This order is the same in both ML methods.

Table S3: Relative contribution of individual features.

Human	Features	Sn	Sp	MCC	ΔMCC
LR	all	86	90	0.77	-
	up trimer	83	88	0.71	-5%
	down trimer	78	78	0.56	-21%
	cleavage dimer	80	78	0.59	-18%
	nucleosome	73	69	0.42	-35%
LDA	all	85	93	0.78	-
	up trimer	83	90	0.73	-5%
	down trimer	77	78	0.55	-23%
	cleavage dimer	79	79	0.58	-20%
	nucleosome	72	68	0.40	-38%

Arabidopsis	Features	Sn	Sp	MCC	ΔMCC
LR	all	91	91	0.82	-
	up trimer	89	90	0.79	-3%
	down trimer	75	74	0.49	-33%
	cleavage dimer	82	81	0.63	-19%
	nucleosome	80	78	0.59	-23%
LDA	all	92	92	0.84	-
	up trimer	89	89	0.78	-6%
	down trimer	75	74	0.49	-35%
	cleavage dimer	83	81	0.64	-20%
	nucleosome	81	76	0.57	-27%

Prediction of larger poly(A) regions

Our results thus far deal only with 600-nt long poly(A) sequences, meaning that the poly(A) site, if exists, is at the midpoint of the sequence. Here, we demonstrate that our model is capable of making prediction for sequence of any length longer than 600 nts. According to our data, the neighboring positions (±2 nts) of a poly(A) site are usually scored high but not for spurious poly(A) sites. Therefore, we imposed a smoothing method to the scores in a 5-nt window. This method calculates the average scores within the window after dropping the highest and the lowest scores. Five hundred real poly(A) sequences, each 2,000 nts long, were sampled randomly from human, and Arabidopsis datasets. The actual poly(A) site is located in the middle of the sequence. The logistic model used a 600-nt window to scan the sequences from left to right to make predictions

regarding whether the midpoint of the window was a poly(A) site or not. Figure S4 shows the results for Arabidopsis. In the upper panel, positions are marked from -700 to +700 along the horizontal axis. The vertical axis represents each of 500 sequences. The model has to make prediction for 1,401 sites per sequence. Altogether, the upper panel contains 700,500 (1,401x500) sites. Predicted poly(A) sites are marked in black in the upper panel. Ideally, only 500 out of 700,500 sites should be predicted as poly(A) sites. The results show that 8.5% of them were predicted as poly(A) sites by the logistic model. The error rate is consistent with the result in Table 3 in the main text. The accumulation of predicted poly(A) sites per position is represented in the lower panel. Similar results were obtained for human data. The error rate is 9%, which is also consistent with the result in Table 3 in the main text.

A

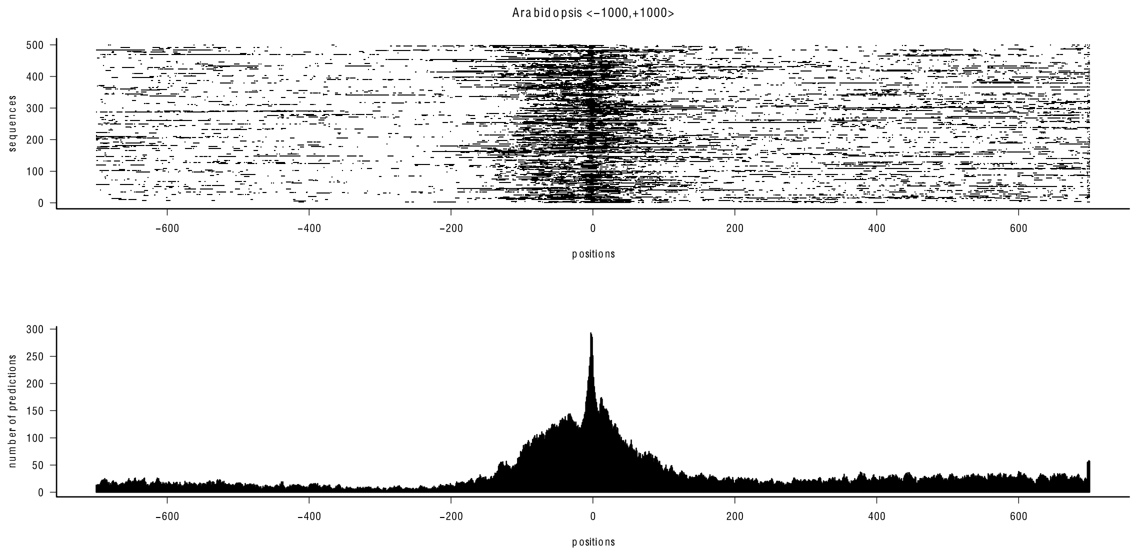

B

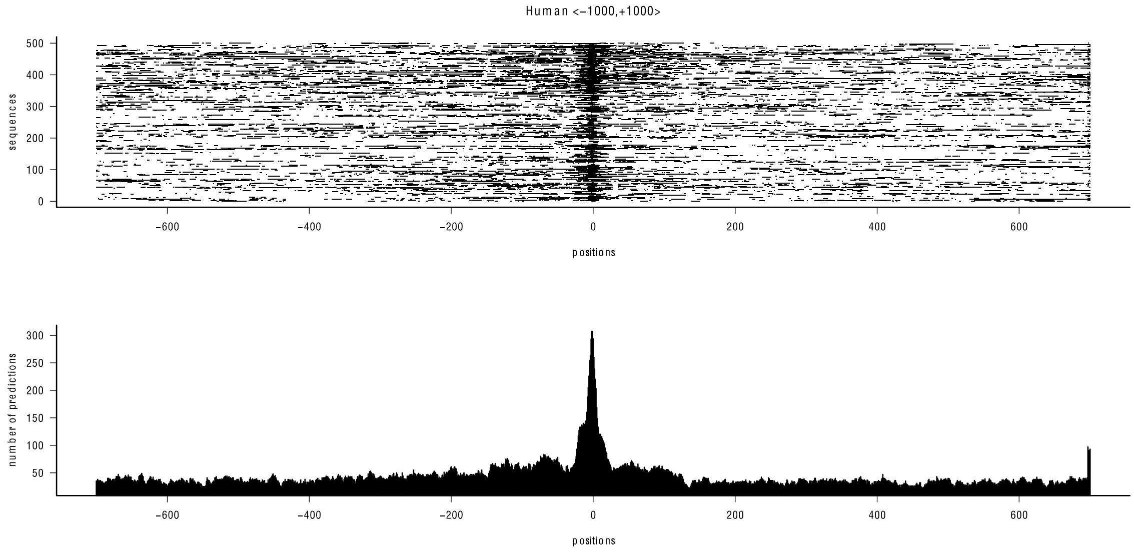

Figure S4: Predictions of five hundred 2,000-nt poly(A) sites by sliding a 600-nt sliding window from left to right. The actual poly(A) site is at position zero or middle of the sequence. The upper panel displays predictions of 500 sequences in region <-700,+700>. Positions predicted as poly(A) sites are marked by black dots. The lower panel accumulates the total number of predicted poly(A) sites by positions. A) Arabidopsis, B) Human.

Cross species PAS predictions

18S

A

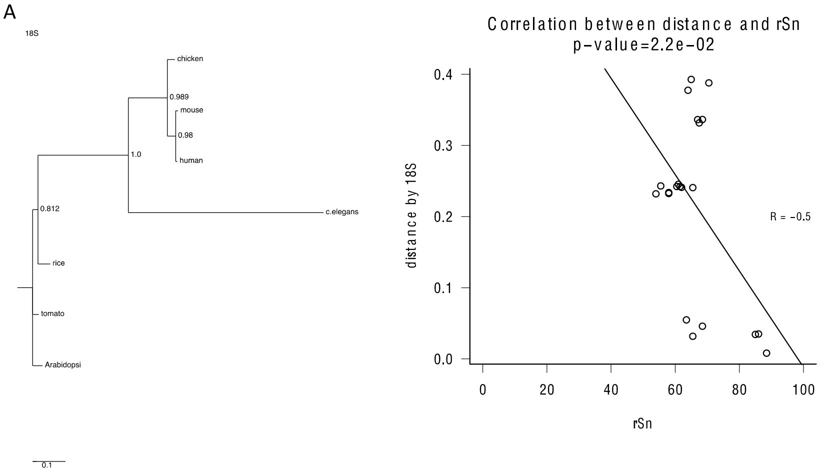

GAPDH

B

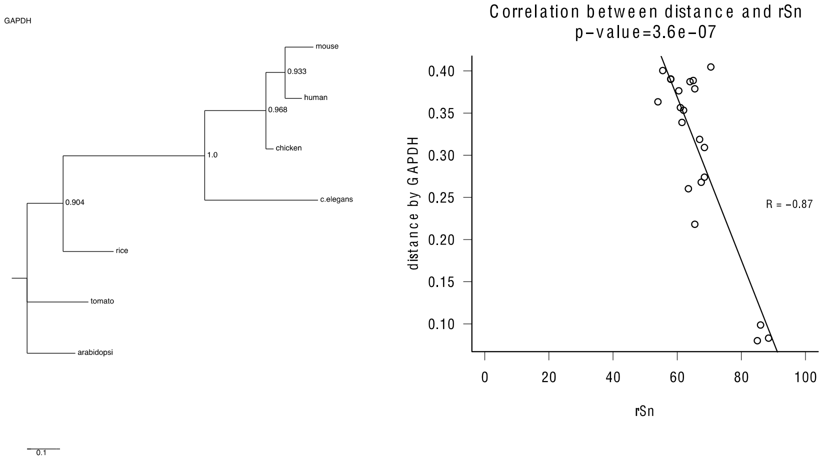

CPSF3

C

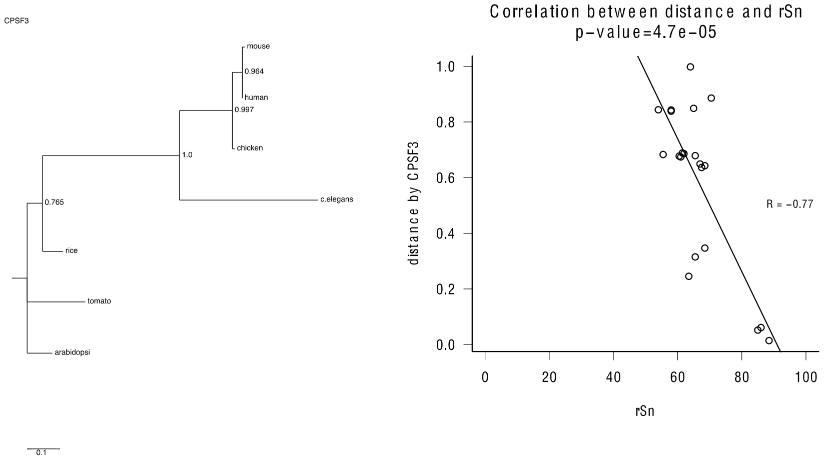

Figure S5. Correlation between phylogenetic distance and reciprocal sensitivity (rSn) between seven species: human, mouse, chicken, c.elegans, rice, Arabidopsis, and tomato. On the left panel of A-C, phylogenetic trees based on ribosomal RNA 18s, GAPDH, CPSF3, respectively. On the right panel, y-axis represents pair-wise phylogenetic distance between a pair of species based on the tree on the left panel. X-axis denotes rSn for a pair of species as defined in the Methods and Materials section in the main text. Twenty-one data points (circles) are plotted in the graph where each represents a pair of different species. Correlation (R) is calculated using Pearson method. P-value of the correlation is stated under the title of the graph on the right panel.

References:

1. Tabaska JE, Zhang MQ: **Detection of polyadenylation signals in human DNA sequences**. *Gene* 1999, **231**(1-2):77-86.
2. Graber JH, McAllister GD, Smith TF: **Probabilistic prediction of Saccharomyces cerevisiae mRNA 3'-processing sites**. *Nucleic acids research* 2002, **30**(8):1851-1858.
3. Legendre M, Gautheret D: **Sequence determinants in human polyadenylation site selection**. *BMC genomics* 2003, **4**(1):7.
4. Liu H, Han H, Li J, Wong L: **An in-silico method for prediction of polyadenylation signals in human sequences**. *Genome informatics International Conference on Genome Informatics* 2003, **14**:84-93.
5. Cheng Y, Miura RM, Tian B: **Prediction of mRNA polyadenylation sites by support vector machine**. *Bioinformatics* 2006, **22**(19):2320-2325.
6. Koh CH, Wong L: **Recognition of polyadenylation sites from Arabidopsis genomic sequences**. *Genome informatics International Conference on Genome Informatics* 2007, **19**:73-82.
7. Tzanis G, Kavakiotis I, Vlahavas I, Bioengineering I, Ieee: **Polyadenylation Site Prediction Using Interesting Emerging Patterns**. *In Proceedings of the 8th Conference on Bioinformatics and Athens Greece* 2008.
8. Ji G, Wu X, Shen Y, Huang J, Quinn Li Q: **A classification-based prediction model of messenger RNA polyadenylation sites**. *Journal of theoretical biology* 2010, **265**(3):287-296.
9. Kalkatawi M, Rangkuti F, Schramm M, Jankovic BR, Kamau A, Chowdhary R, Archer JAC, Bajic VB: **Dragon PolyA Spotter: predictor of poly(A) motifs within human genomic DNA sequences**. *Bioinformatics (Oxford, England)* 2012, **28**(1):127-129.
10. R Development Team: **R: A language and environment for statistical computing**. *R Foundation for Statistical Computing, Vienna, Austria* 2011.
